# Preserving the woody plant tree of life in China under future climate and land-cover changes

**DOI:** 10.1098/rspb.2022.1497

**Published:** 2022-12-07

**Authors:** Shijia Peng, Ruocheng Hu, Santiago José Elías Velazco, Yuan Luo, Tong Lyu, Xiaoling Zhang, Jian Zhang, Zhiheng Wang

**Affiliations:** ^1^ Institute of Ecology and Key Laboratory for Earth Surface Processes of the Ministry of Education, College of Urban and Environmental Sciences, Peking University, Beijing 100871, People's Republic of China; ^2^ Center for Nature and Society, School of Life Sciences, Peking University, Beijing 100871, People's Republic of China; ^3^ Instituto de Biología Subtropical (IBS), Universidad Nacional de Misiones (UNaM)—Consejo Nacional de Investigaciones Científicas y Técnicas (CONICET), Misiones, Argentina; ^4^ Programa de Pós-Graduação em Biodiversidade Neotropical, Universidade Federal da Integração Latino-Americana, Foz do Iguaçu, Brazil; ^5^ Department of Botany and Plant Sciences, University of California—Riverside, Riverside, CA, USA; ^6^ Zhejiang Tiantong Forest Ecosystem National Observation and Research Station, School of Ecological and Environmental Sciences, East China Normal University, Shanghai 200241, People's Republic of China

**Keywords:** conservation priority, effectiveness, phylogenetic spatial prioritization, post-2020 framework, protected areas, tree of life

## Abstract

The tree of life (TOL) is severely threatened by climate and land-cover changes. Preserving the TOL is urgent, but has not been included in the post-2020 global biodiversity framework. Protected areas (PAs) are fundamental for biological conservation. However, we know little about the effectiveness of existing PAs in preserving the TOL of plants and how to prioritize PA expansion for better TOL preservation under future climate and land-cover changes. Here, using high-resolution distribution maps of 8732 woody species in China and phylogeny-based Zonation, we find that current PAs perform poorly in preserving the TOL both at present and in 2070s. The geographical coverage of TOL branches by current PAs is approx. 9%, and less than 3% of the identified priority areas for preserving the TOL are currently protected. Interestingly, the geographical coverage of TOL branches by PAs will be improved from 9% to 52–79% by the identified priority areas for PA expansion. Human pressures in the identified priority areas are high, leading to high cost for future PA expansion. We thus suggest that besides nature reserves and national parks, other effective area-based conservation measures should be considered. Our study argues for the inclusion of preserving the TOL in the post-2020 conservation framework, and provides references for decision-makers to preserve the Earth's evolutionary history.

## 1. Background

The ongoing climate and land-cover changes have led to a sharp rise in the extinction of global biodiversity to a level that is much higher (approx. 100 times) than the natural background [1,2]. Globally, more than 200 000 protected areas (PAs) covering roughly 17% of the Earth's lands have been established to halt biodiversity loss [3]. However, most PAs have been created without considering the impacts of future climate and land-cover changes on species distribution and biodiversity [4,5]. Consequently, the relevance of existing PAs for protecting biodiversity under climate and land-cover changes remains largely unknown. Moreover, the zero- and first-drafts of post-2020 conservation framework of the Convention on Biological Diversity (CBD) propose to expand global PAs to cover at least 30% of terrestrial areas by 2030 [6]. Thus, the spatial prioritization of PA expansion under future climate and land-cover changes represents a current challenge.

In conservation planning, species richness (SR) has been widely used to identify conservation priorities in previous studies. Evolutionary history across the tree of life (TOL), an important facet of biodiversity and normally measured by phylogenetic diversity (PD), has also been shown to be relevant to biodiversity conservation [7]. Theoretical and empirical studies indicate that PD is positively related to the feature diversity (i.e. the amount of species features) [8–10], and option values (i.e. the potential unanticipated needs that future generations may be able to benefit from biodiversity) in a region and community [11,12]. Moreover, PD has been found to have strong contributions to the ecosystem functions and services, and hence represents the ‘national heritage' for the benefit of humanity [12–14]. It is noteworthy that the TOL is being pruned at a fast rate due to strong impacts of global changes and human disturbances [15,16], leading to a reduction in the completeness of the TOL and rapid loss of evolutionary history and total PD. Therefore, maximizing the conservation of the TOL is increasingly recognized as a high priority [17,18], and requires systematic identification of conservation priorities based on the TOL rather than traditional, species-based prioritization approaches [19–21]. However, preserving the TOL has not yet been clearly included in most international biodiversity conservation frameworks such as the first draft of the global post-2020 conservation framework [6]. Upgrading the PA network to better preserve the TOL by future PA expansion is an urgent need for global biodiversity conservation.

China is one of the world's mega-biodiverse countries, and hosted the 15th CBD conference in late 2021. However, China has experienced dramatic climate and land-cover changes in the past, which are likely to continue in the future. Therefore, China is one of the most important countries for achieving the global biodiversity conservation targets. Our study focused on woody species (i.e. species with a prominent above-ground stem that could be persistent through time and changing conditions) in China, as they are a fundamental component of forest ecosystems and provide habitats for high trophic levels [22,23] and multiple ecosystem services for human well-being (e.g. climate regulation, biomass production and pollination).

Here, by integrating the high-resolution distribution maps and phylogenies of 8732 woody species in China with species distribution models (SDMs) and systematic conservation planning approaches, we evaluated the effectiveness of the existing PAs to conserve the TOL of woody species in China, and identified the priority areas for future PA expansion that can better preserve the TOL under different scenarios of climate and land-cover changes. Specifically, we aim to ask: (i) how effective the existing PAs are in preserving the TOL of woody species in China under current and future climate and land-cover conditions; (ii) which regions should be prioritized for the conservation of TOL under future climate and land-cover scenarios; and (iii) how much more of the TOL of woody species in China could be protected by the identified priority areas under different conservation goals.

## 2. Material and methods

### (a) Species distribution data

The county-level distribution data of all 11 405 woody plants in China were taken from the *Atlas of Woody Plants in China: Distribution and Climate* [24,25]. Then the species distributions were substantially updated using recently published national and regional floras and specimen records (see full list in electronic supplementary material, appendix S1). The county-level distribution maps were then transformed into an equal-area grid with a spatial resolution of 20 × 20 km to eliminate the impacts of area on the estimates of species diversity. To improve the accuracy of plant species distributions in the transformation, we used species' elevation ranges and habitat types compiled from national and provincial floras to refine their distributions within each county (see electronic supplementary material, appendix S2 for more details about the data and methods for the transformation from county-level distributions to gridded distributions). After removing grid cells with less than 200 km^2^ on the country borders and along the coast line, 23 718 grid cells remained. Since the performance of SDMs depends on the sample size of species distributions, we only included species with at least 20 occurrences, totalling 8732 species.

### (b) Environmental data

In SDM calibration, we used six bioclimatic and six land-cover variables to represent current and future environmental conditions. Current climate data (1960–1990) with a spatial resolution of 2.5 arc min were obtained from the WorldClim v.1.4 (https://worldclim.org/). The six climate variables were temperature seasonality (bio4), mean temperature of the warmest (bio10) and the coldest quarters (bio11), precipitation seasonality (bio15), and precipitation of the wettest (bio16) and the driest quarters (bio17). These variables have low multicollinearity (|r| < 0.7) and have clear ecological relevance to woody species distributions following previous studies [5]. The climate data were upscaled to 20 × 20 km resolution by averaging values of finer cells (i.e. 2.5 arc minutes) within each 20 × 20 km grid cell. Future climate data were also obtained from the WorldClim database. To account for the potential uncertainties in SDM projections induced by different general circulation models (GCMs), we adopted future climate data projected by five GCMs: BCC-CSM1-1.1, MRI-CGCM3, CCSM4, GISS-E2-R and IPSL-CM5A-LR (see electronic supplementary material, appendix S2 for the selection of GCMs). For each GCM, projections based on two representative concentration pathways (i.e. RCP 2.6 and RCP 8.5) were used.

Previous studies indicate that changes in land-cover may influence species distributions [5,16,26]. We therefore included land-cover data in the calibration and projection of SDMs. Specifically, current and future land-cover data with a 1 km resolution were obtained from Li *et al*. [27], including five land cover types (i.e. forest, grassland, farmland, urban and barren). These data provide information on the consequences of anthropogenic changes to the Earth's surface over time. It is noteworthy that the future projection of land-cover types was generated under the earlier emission scenarios (i.e. A1B, A2 and B1). Following IPCC [28], we matched the B1 and A2 scenarios with the RCP 2.6 and RCP 8.5, respectively. We calculated the proportion of each land-cover type within each grid cell, and adopted each as an independent predictor of SDMs (see more details in electronic supplementary material, appendix S2). Soil variables were not included since recent study indicated that climatic variables are more important than soil for simulating woody species distributions in China [5].

### (c) Phylogeny of woody species

A recently published genus-level phylogeny of seed plants in China was used [29], which covers 1092 genera of Chinese woody species. To build a species-level phylogeny containing woody species in China, we inserted the woody species of each genus into the phylogeny as polytomies, and randomly resolved the polytomies within each genus by a birth–death model using the R function ‘sticktips’ [30]. To evaluate the potential influences of random topologies on our results, we generated 100 randomly resolved trees, and repeated the following analyses with trees.

### (d) Protected areas

PAs in China were from Zhang *et al.* [31] (see electronic supplementary material, figure S1 in appendix *S3*). We included only the provincial and national PAs because (i) they are better managed than most prefectural and county-level PAs, and (ii) their boundaries are better maintained. We further excluded 30 PAs in the ‘Mangroves biome' (i.e. marine PAs). A total of 1161 PAs were finally retained. By overlaying the PA polygons with the 20 × 20 km^2^ grid cells, we estimated the proportion of area covered by PAs in each grid cell and considered a cell to be protected if PA polygons covered greater than 30% of its area. We used a 30% cut-off following the zero and first drafts of the post-2020 conservation framework [6].

### (e) Data on human pressures

We used the recently published human modification index (HMI) with a spatial resolution of 300 m to characterize human pressures [32]. HMI is defined as the human stressors or processes that have caused, are causing and may cause severe impacts on biodiversity and ecosystems. This index synthesized 14 global-scale stressor layers, including built-up, croplands and pasture-lands, grazing, oil and gas production, mining and quarrying, renewable and non-renewable power generation, roads, railways, power lines, electrical infrastructure, logging and wood harvesting, human intrusions, reservoirs and air pollution. Globally, HMI ranges from 0 to 1. The HMI of a 20 × 20 km grid cell was calculated as the average of all data points within it. Following [32], we classified HMI into three categories representing different human pressure intensities: low (mean HMI < 0.1), moderate (0.1 ≤ HMI < 0.4) and high (HMI ≥ 0.4).

### (f) Species distribution models

We used five SDM algorithms encompassing a variety of statistical techniques for modelling species distributions: classification tree analysis, generalized linear model, generalized boosting model, random forest and maximum entropy (see electronic supplementary material, appendix S2 for the detailed set-up of each SDM algorithm). For each species, the models were trained with randomly selected 80% of the distribution data, and tested with the remaining 20% using the true skill statistic (TSS) [33]. We repeated this procedure 10 times for each species. Only models with TSS ≥ 0.5 were used in the following analyses [34,35] (electronic supplementary material, figure S2 in appendix S3). Then the retained models were used to predict species distributions at 20 × 20 km^2^ resolution under current and 2070s conditions. Median ensemble forecasts were performed for each species across all GCM and SDM combinations (i.e. totally 25 combinations) and the outputs were reclassified into binary maps using the threshold that maximizes the TSS [35]. We also predicted binary species distributions and conducted all the analyses for each GCM and SDM combination to explore model uncertainties. All SDM models were conducted in the BIOMOD2 package in R 4.2.1 [36].

The spatial extent for all SDM calibrations and predictions was the terrestrial range of China [5]. SDMs often overpredict suitable areas of species, which may adversely bias the spatial prioritization for conservation [37]. We thus adopted a buffered minimum convex polygon surrounding species occurrences to constrain modelled current species distributions [38]. We used a 200 km buffer width following previous studies [5,16].

Dispersal may significantly influence the responses of species distributions to global changes, and hence should be considered in simulating species distributions [39]. Here, we projected species distributions under future climate and land-cover change with three dispersal scenarios: (i) full dispersal, (ii) 20 km per decade and (iii) no dispersal. The second dispersal scenario was conducted by applying a 200 km (20 km per decade from the 1970s to the 2070s) buffer surrounding the modelled current distribution range of each species to its projected future distribution [40].

### (g) Data analyses

#### (i) The vulnerability of species and phylogenetic branches

The vulnerability of species and phylogenetic branches to future climate and land-cover changes was evaluated using relative changes in their total suitable habitats (CSH) (see more details in electronic supplementary material, appendix S2). A branch was considered present in a given grid cell if any of its descendants occurred in that cell. We then assessed the relationship between current range sizes of species (or branches) and CSH. For species-level analysis, we further obtained the IUCN Red List categories of all 8732 species and compared the effects of current range sizes and IUCN Red List categories of species on their CSH under future scenarios.

#### (ii) Gap analysis

Following Rodrigues *et al.* [41], we identified gap species and branches by overlaying the distribution of each species (or branch) with the PA map. Based on the conservation target of each species (or branch) and the percentage of each species (or branch)’ geographical range covered by PAs, we classified species (or branches) into four groups under current and different future climate and dispersal scenarios (see details in electronic supplementary material, appendix S2): (i) unprotected: species’ (or branches) range is completely outside PAs; (ii) gap: up to 20% of the conservation target; (iii) partial gap: 20–90% of the conservation target and (iv) covered: greater than 90% of the conservation target.

#### (iii) Phylogeny-based spatial prioritization

We first conducted phylogeny-based spatial prioritization analysis using Zonation 4.0 [42] to identify priority areas best representing the entire TOL of Chinese woody species under both current and future conditions. Zonation identifies priority areas in a landscape mainly based on the representation of biodiversity features (i.e. branches in our study) and feature weights (branch length). We calculated the occurrence of each branch of the phylogeny as input layers of Zonation, and assigned each branch a weight that was proportional to its branch length, indicating the relative importance of a given branch in meeting the conservation goal [19,20]. Using these basic conservation values, we calculated the marginal loss of each grid cell in each iteration, and the least value was iteratively removed until all grid cells are removed. Several algorithms could be used to estimate the marginal loss including core-area zonation (CAZ), which removes grid cell based on the (weighted) maximum value across all features in a cell, and additive benefit function (ABF), which sum conservation values in a cell [42]. We used both algorithms, and only presented CAZ-based results in the text for the following reasons. First, preliminary analyses using the *band collection statistics* tool in ArcGIS 10.3 showed high correlation coefficients (*r* > 0.7) between the maps of priority areas identified by the two algorithms. Second, the aim of this study was to maximize the conservation of overall Chinese TOL of woody plants, and protecting long branches (i.e. the maximum value in a cell) represents the greatest gain in the TOL. Therefore, CAZ algorithm is theoretically more favourable than ABF in our analysis [19,20].

With phylogeny-based Zonation analysis, we identified the ideal conservation areas to optimize the representation of TOL of woody species in China under the current and future environmental conditions as the top 30% (i.e. the target PA coverage by 2030 of the post-2020 conservation framework) or 50% (i.e. E. O. Wilson's ‘Half-Earth' proposal [43]) of the grid cells that capture the highest proportion of the total PD of woody plants in China [43,44]. By comparing the current and future ideal conservation areas, we identified (i) the overlapped ideal conservation areas under the current and future environmental conditions; (ii) the current ideal conservation areas that will disappear in the future; and (iii) newly formed ideal conservation areas in the future. To account for the phylogenetic uncertainty, we ran 100 Zonation and assessed the similarities of the 100 priority layers under current conditions using the *band collection statistics* tool in ArcGIS 10.3. Preliminary analyses showed that the average Pearson's correlation coefficient (*r*) between each combination of two input priority maps is 0.98. This means that the phylogeny randomization has no significant impacts on our results (see electronic supplementary material, appendix S4). We thus presented the findings from a single phylogeny in the main text for simplicity.

#### (iv) The effectiveness of current protected areas in protecting tree of life

To assess the performance of the current PAs in preserving the TOL in China, we overlaid three types of ideal conservation areas with the PA layer to calculate the coverage of different ideal conservation areas by existing PAs. To further confirm the validity of our conclusions, we also calculated the PD of woody species in each grid cell (i.e. local PD estimation) following the definition of Faith [8] (electronic supplementary material, appendix S2). All grid cells were divided into five classes according to their local PD values. We estimated the proportions of protected and unprotected grid cells within each PD class under current and 2070s conditions.

#### (v) Identification of future areas for protected area expansion

In order to identify the regions for expansion of current PAs that could best preserve the entire TOL of woody species in China under future climate and land-cover changes, we further conducted analyses with current PA layer as a mask in Zonation (i.e. established PAs are defaulted to high priority) [42]. Since current national and provincial PAs accounted for approximately 15% of land areas in China, we identified an additional 15% and 35% land areas beyond the current PAs, corresponding to the conservation target of 30% or 50% PA coverage, respectively. We estimated the geographical coverage of each branch in the phylogeny of woody species and the regions with different PD values by the current PAs and the targeted future expansion areas to evaluate whether the identified PA expansion areas could improve the conservation of the TOL in China. We also compared the human pressures in the current PAs, the selected PA expansion areas and unprotected regions (termed as non-PAs).

To evaluate the gains from the phylogeny-based spatial prioritization in preserving the TOL of woody species in comparison with traditional approaches considering only SR, we further conducted species-based spatial prioritization using Zonation. We compared the priority areas for Chinese woody species conservation by phylogeny and species, and then compared the mean geographical coverage of all branches by the 30% and 50% priorities between phylogeny and species-based approaches using Student's *t-*test.

To assess the conservation status of regions with novel (non-analogous) climate, we conducted area of applicability (AOA) analysis to identify the areas with novel climate for species distributions. AOA was estimated using the dissimilarity between current and future climates in the multidimensional space of climate factors [45]. We then overlapped the map of novel-climate regions with priority areas for PA expansion to determine the novel-climate areas not covered by PAs.

## 3. Results

We find approximately 21.7% of species (13.4% branches) will lose their suitable areas, while approximately 40% of species (32% branches) will greatly expand (CSH > 80%) their areas under the most positive scenario (i.e. full dispersal and RCP 2.6). Under the most severe scenario (i.e. no dispersal and RCP 8.5), 7.2% species (3.7% branches) are projected to go local extinctions (electronic supplementary material, figures S3 and S4 in appendix S3). The vulnerability of species and branches increases with the decrease in their current range size (electronic supplementary material, table S1 in appendix S3), and species (or branches) loss is most serious in warm temperate deciduous coniferous forest and subtropical evergreen broad-leaved forest regions (electronic supplementary material, figures S5 and S6).

According to the conservation targets of each species (or branch), we identify 35.4% (17.1%) gap species (branches), while 10.4% (15.3%) species (branches) are fully covered by PAs and 50% (61.6%) are partial gap species (branches) under current conditions. The number of gap species (branches) under future and land-cover changes depends on different dispersal scenarios. Under the full dispersal and RCP8.5 scenario, the proportion of gap species (branches) will slightly decrease due to range expansions of some species. By contrast, under the no dispersal scenario, the proportion of gap species (branches) will increase compared with current conditions, with 38.8% gap species and 24.8% gap branches under RCP8.5 scenario (electronic supplementary material, figures S7 and S8).

**Figure 1.**
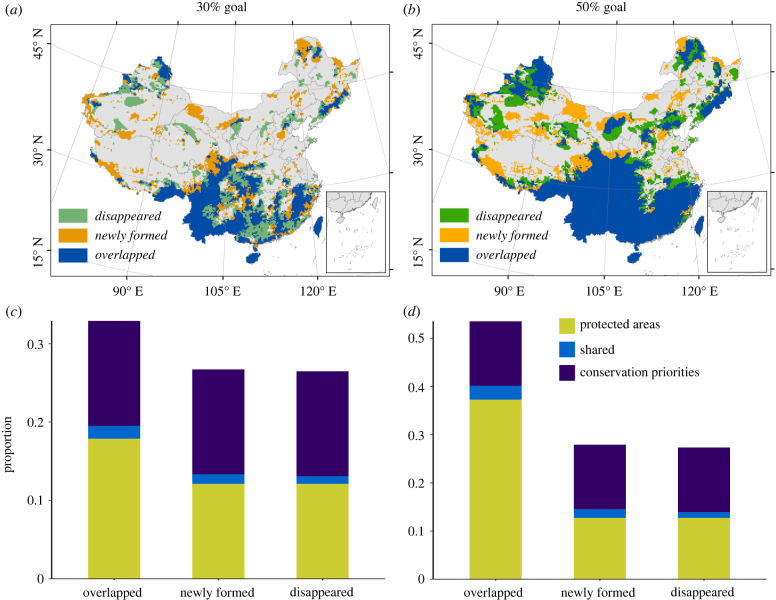
(*a*,*b*) Geographical patterns of the top (*a*) 30% and (*b*) 50% conservation priorities under current and future environmental conditions. The disappeared, newly formed and overlapped priorities from the current to the future conditions are shown in different colours. (*c*,*d*) The percentages of the existing protected areas, the three types of the conservation priorities and their coverage by existing protected areas (i.e. shared areas) for (*c*) 30% and (*d*) 50% conservation goals. See electronic supplementary material, table S2 in appendix S3 for more details. Disappeared: current priority areas that will disappear in the future; overlapped: priority areas under both current and future conditions; newly formed: newly formed priority areas in the future. These priority areas are determined using the geographical distribution of each branch in Zonation under both current and future conditions. Here, the future distribution is estimated under RCP 8.5 and full dispersal scenarios.

The ideal conservation areas for best preserving the TOL identified under the current and future climate and land-cover conditions are largely overlapping, and are mainly located in the Hengduan Mountains, the Yunnan-Guizhou Plateau, the Qinba mountainous region and the hilly areas of southeastern China (figure 1*a,b*; electronic supplementary material, figures S9 and S10). Interestingly, we find that some areas not identified as ideal conservation areas under the current conditions will become new ideal conservation areas under future environmental conditions, including the southern margin of the Qinghai-Tibet Plateau, the Daxinganling Mountains and the Changbai Mountains. It is noteworthy that the current PAs protect less than 3% of the identified ideal conservation areas (i.e. areas shared between PAs and the identified ideal conservation areas), no matter whether the ideal conservation areas are identified to cover 30% or 50% of terrestrial lands (electronic supplementary material, table S2; figure 1*c*,*d*).

The current spatial patterns in local PD are generally consistent with those under future environmental conditions (electronic supplementary material, figures S11–S13). Local PD is highest in southern China, but lowest in the Qinghai-Tibet Plateau and drylands in northwest China. Future climate and land-cover changes would lead to an increase in local PD in the southern margin of the Qinghai-Tibet Plateau and the Hengduan Mountains, but a decrease in local PD in mountainous regions of southern China (electronic supplementary material, figure S14). The local PD increases with the increase in annual mean temperature, annual mean precipitation, elevation and natural vegetation coverage, but decreases with the temperature seasonality (electronic supplementary material, table S3). The results on the conservation of local PD suggest low effectiveness of current PAs in protecting the TOL of woody plants in China. Most of the PAs are located in regions with relatively low local PD, while regions with high local PD are largely unprotected, regardless of current or future climate and land-cover conditions (electronic supplementary material, figures S11–S13).

**Figure 2.**
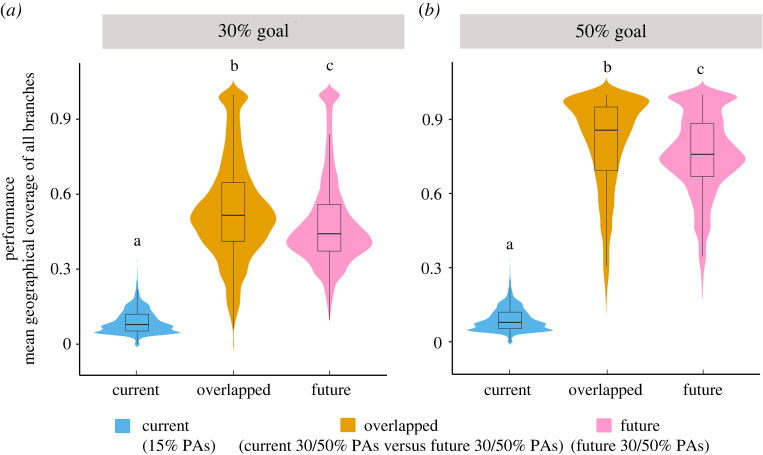
Violin plots of the mean geographical coverage of all branches by the existing protected areas (PA; approx. 15% of land area; blue), the overlapped conservation priorities between the current and future environmental conditions (orange) and the future conservation priorities (pink) under the (*a*) 30% and (*b*) 50% conservation targets. The mean geographical coverage of all branches by the existing protected areas is the same for both conservation targets. Different letters indicate significant differences based on Tukey's honest significant difference test (*p* < 0.05). Here, the future species distribution is estimated under RCP8.5 and full dispersal scenarios.

**Figure 3.**
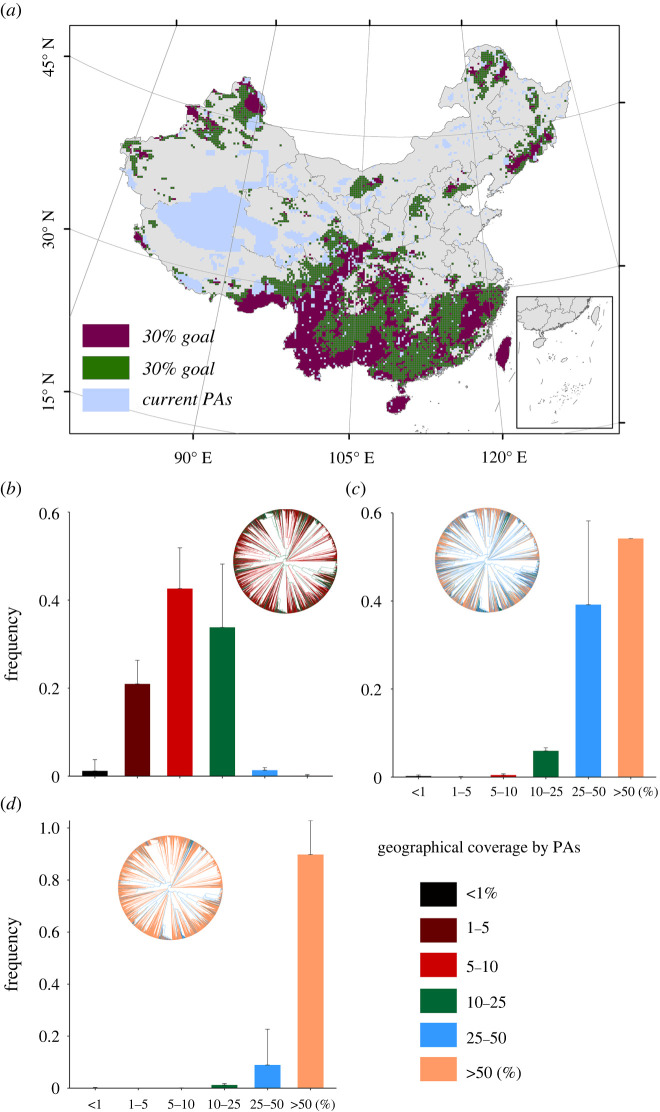
(*a*) Regions for future protected area (PA) expansion. (*b–d*) The status of current protection (*b*) and potential conservation gains for the tree of life of woody plants based on different PA expansion scenarios: (*c*) 30% conservation goal and (*d*) 50% conservation goal. For each branch of the phylogenetic tree, the protection level is calculated as the geographical coverage (percentage of total branch occurrences) by protected areas: (*b*) 15% existing PAs; (*c*) 15% existing PAs + 15% PA expansion areas; (*d*) 15% existing PAs + 35% PA expansion areas. Histograms show the percentage of phylogenetic branches for different protection classes. Spatial pattern of future PA expansion areas is mapped based on the ensembled species distributions. Error bars in histograms represent uncertainty (i.e. variation results) originating from the different SDM and GCM combinations. Colours close to orange suggest a higher degree of protection. Here, the future species distribution is estimated under full dispersal and RCP 8.5 scenarios.

**Figure 4.**
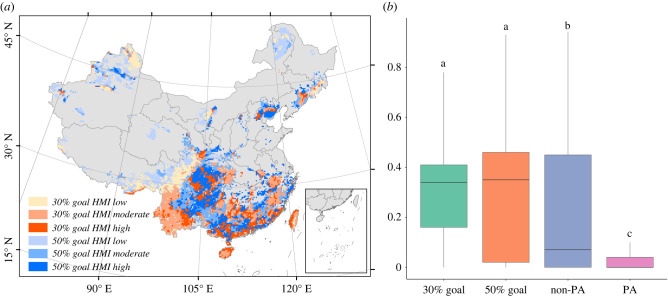
(*a*) Human modification index (HMI) for the top 30% and 50% protected area expansion. (*b*) The difference in HMI among these areas, non-protected areas (non-PAs) and existing protected areas (PAs). Different letters indicate significant difference based on Tukey's honest significant difference tests (*p* < 0.05). Here, the future species distribution is estimated under RCP 8.5 and full dispersal scenarios.

Currently, the mean geographical coverage of all branches by existing PAs is 9%. However, the effectiveness in conserving the TOL of woody plants in China can be substantially improved by expanding PAs in the overlapped priority areas under both current and future conditions (figure 2; electronic supplementary material, figures S15 and S16). Specifically, the mean geographical coverage of all phylogenetic branches will be improved from 9 to 52% by an increase of 15% in PA coverage (i.e. to reach 30% PA coverage in total), and to 79% by an increase of 35% in PA coverage (i.e. to reach 50% PA coverage in total) (figure 3; electronic supplementary material, figures S17 and S18). Meanwhile, the effectiveness in protecting local PD could also be improved by expanding PAs. The regions with the highest local PD are largely protected after PA expansion in the overlapped priority areas (electronic supplementary material, figure S19). It is noteworthy that the mean HMI is higher in the identified priority areas for PA expansion than in the existing PAs and non-PAs (figure 4; electronic supplementary material, figures S20 and S21; one-way ANOVA, *p* < 0.01). Approximately one-third of the identified priority areas for PA expansion are suffering from very high human pressure, including the Yunnan-Guizhou Plateau, northwest Hainan Island and Taiwan Island.

**Figure 5.**
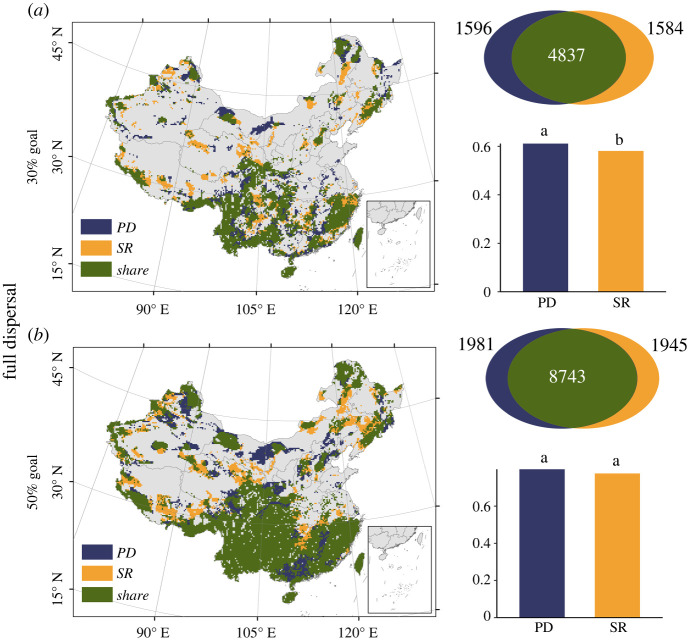
Comparing the conservation priorities for woody species conservation by maximizing phylogenetic diversity (PD) and SR, constrained to (*a*) 30% and (*b*) 50% conservation goal of total land area. Dark blue: conservation priorities determined by phylogeny-based prioritization. Yellow: conservation priorities determined by species-based prioritization. Dark green: shared conservation priorities. The Venn plots show overlapping and unique areas for prioritization based on PD and SR. Numbers in Venn plots are the number of grid cells of three types of priorities. Histograms represent the mean geographical coverage of all branches by 30% (or 50%) PD priorities and SR priorities. Dissimilar letters within each histogram indicate significant difference between two groups from Student *t-*test (*p* < 0.05). Here, the future species distribution is estimated under full dispersal and RCP 8.5 scenarios.

We find differences between the conservation priorities identified by the species- and phylogeny-based prioritization (figure 5; electronic supplementary material, figure S22). Some areas are recognized to be important for the conservation of the TOL of woody plants in China, but are overlooked in the species-based analysis. These areas are mainly distributed in Nanling mountain, the hilly regions of southeastern China, Alxa Plateau, the northern margin of Greater Khingan and the western margin of Hengduan Mountain. Moreover, under the 30% conservation goal, the mean geographical coverage of all phylogenetic branches by the phylogeny-based priority areas is significantly higher than that by the species-based priority areas.

Most of locations with novel climate are distributed in the northern mountainous areas, including Tianshan Mountain, Altai Mountain, Qilian Mountain and Kunlun Mountain. These novel-climate regions are not covered by the identified PA expansion areas (electronic supplementary material, figure S23).

## 4. Discussion

Effectively preserving the TOL is increasingly recognized as an important conservation target [17]. Here, we comprehensively evaluate the effectiveness of the existing PA network for preserving the TOL of woody plants in China. We find that the existing PAs in China are ineffective in preserving the TOL under both the current and future climate and land-cover conditions. Luckily, we demonstrate that strategic expansion of PAs could significantly improve the conservation status of the TOL of woody plants in China. Our findings here provide a clear guide to spatially prioritize the conservation of the Chinese TOL under the post-2020 biodiversity conservation framework.

Due to limited conservation funding, and difficulties in land acquisition and management, most spatial planning of PAs largely depends on local socio-economic conditions and histories rather than biodiversity consideration [46]. Most PAs are currently located in economically less developed regions to reduce the cost of conservation. Although the number and coverage of PAs have significantly increased in China over the past three decades [47], the current spatial distribution of PAs is far from optimal, and falls short of being ‘phylogenetically representative' (figure 1; electronic supplementary material, figures S9–S13). For instance, most large PAs are in western China, where the human footprint is low and the climate is dry and cold. By contrast, southern and southwestern China are much less covered by the existing PA network, and the PAs in these regions are spatially sporadic, and poorly interconnected [48]. This biased distribution of PAs has primarily led to the low effectiveness in preserving the local PD and the TOL of woody species in China. Therefore, a better strategic planning of PA expansion is urgently needed to halt the decline of evolutionary history under future climate and land-cover changes.

Our study identified the priority areas for PA expansion to meet different conservation goals. The identified priority areas under the current and future climate and land-cover conditions are largely overlapping, and these areas represent the most effective target regions for PA expansion to preserve the plant TOL in China (figures 3; electronic supplementary material, figures S15–S18). Expansion of PAs in these identified priority areas to reach 30 or 50% land coverage could increase the average PA coverage of the branches in Chinese TOL from the current level to 52% and 79%, respectively (figures 2 and 3). These results demonstrate that the identified priority areas for PA expansion are highly efficient for preserving the Chinese TOL of woody plants, and could be used in future practice of PA expansion. Interestingly, we find that some currently unsuitable areas will become suitable for PA expansion under future environmental conditions. Since these new areas are likely to be important for future persistence of woody plants in China, they should be given conservation attention in future PA expansion.

Notably, greater than 50% of the identified priority areas for PA expansion are experiencing moderate to high human pressures (figure 4; electronic supplementary material, figures S20 and S21), suggesting that future PA expansion programs may be costly, and the expected conservation goals may be hard to achieve. According to a recently released document (see https://www.gov.cn/zhengce/2019-06/26/content_5403497.htm), China will establish different PAs including the governance of government, strict nature reserves and national parks [49]. In the identified priority areas for PA expansion that have high human pressures, it may be quite cost-ineffective and challenging to construct these types of PAs. To achieve the future 30% or 50% conservation goals, it is necessary to pay more attention to other effective area-based conservation measures (OECMs) such as fengshui forests (i.e. small remnant forest patches coexisting with natural villages) [50,51]. Although OECMs do not meet the IUCN definition of PAs, they may play important and complementary roles in conservating biodiversity outside the PA network [52,53], especially in eastern China where PAs are sporadic and are costly to establish. Previous studies also suggest that the unprotected biodiversity hotspots are normally covered by potential OECMs [54]. Therefore, future restoration or conservation programmes may be preferentially carried out in these easily overlooked areas to preserve biodiversity. Yet well-designed and effective corridors connecting these OECMs are necessary for them to achieve good conservation goals.

It is noteworthy that the Chinese government has made great efforts to promote biodiversity conservation in China recently. Several PA reforms have been implemented, including restructuring government agencies, establishing a national park administration and separation of management from monitoring and supervision [55]. Moreover, the degraded environment has begun to recover due to implementation of national and provincial conservation and restoration programmes [56]. These conservation practices may shed light on the improvement of conservation effectiveness in other countries.

Previous studies show that SR represents a surrogate of PD in conservation planning [57]. However, our results suggest that the priority areas for PA expansion identified by the phylogeny-based prioritization analysis differs from those identified by the species-based approach. The traditional species-based prioritization is much less effective in identifying conservation priorities for protecting the TOL than the phylogeny-based approach (figure 5; electronic supplementary material, figure S22). The existing international biodiversity conservation frames (e.g. post-2020 biodiversity framework) mostly focus on taxonomic diversity rather than evolutionary diversity. The preservation of the TOL has not yet been officially embedded in these conservation frames. Therefore, we suggest that preserving the TOL should be separately considered in future conservation frames.

Several issues may induce uncertainties in our results. First, our spatial prioritization for conserving the TOL is only based on biological components, and lacks the consideration of socio-economic or political factors. For example, some priority areas occur in provinces with low GDP (e.g. southwestern China), which may lead to conflicts between conservation and economic development [39]. We did not evaluate the economic costs of expanding current PAs here. Including these socio-economic factors as cost-layer in Zonation may be advisable to optimize conservation planning. Second, our study identified several novel-climate regions, which are not covered by identified PA expansion areas (electronic supplementary material, figure S23). Since we do not know how species respond to these novel-conditions, decision-makers should pay attention to these novel-climate regions in future conservation. Third, SDMs do not consider the evolution of species adaptation in response to global change, which may overestimate the adverse impacts of global change [58], especially for novel-climates. New methods incorporating adaptation evolution in response to global change are needed to evaluate the contributions of such process to local extinctions. Fourth, our study did not include IUCN information as weight in phylogeny-based prioritization analyses due to difficulty assigning weights to a branch with descendants with different conservation status. Future studies should develop an integrative index incorporating IUCN information in phylogeny-based prioritization.

Our study also points out a few important future research directions. First, species range changes (especially expansion) induced by global change may lead to decreases in beta-diversity and hence biotic homogenization [59,60]. Therefore, adequately exploring the beta-diversity patterns within PA network are important to promote future conservation planning. Second, the geographical patterns in plant diversity may differ from those of other groups (e.g. amphibians, reptiles). Whether the identified priority areas for the conservation of TOL of plants also serve as priority areas for TOL conservation for other groups remains to be evaluated. Last, our study only focused on the Chinese TOL of woody species. For some internal branches found elsewhere in the world, future efforts compiling and refining the distributions and phylogenies for species outside China are needed to conduct spatial prioritizations for the conservation of the angiosperms TOL at larger scales.

## 5. Conclusion

In summary, we find that the current PA network is ineffective in preserving the TOL of woody species in China under both current and 2070 environmental conditions. To improve the preservation of the TOL of woody plants in China, we need to look beyond existing PAs and strategically expand new PAs in the Hengduan Mountains, Yunnan-Guizhou Plateau, Qinba mountainous areas and hilly areas of southeastern China. Due to strong human pressures in the identified priority areas for future PA expansion, we suggest that OECMs such as fengshui forests should be considered to improve the preservation of the TOL of woody plants in China. Our study argues for the inclusion of preserving the TOL in the post-2020 global biodiversity framework, and provides a case for decision-makers to preserve the Earth's evolutionary history.

## Data Availability

The data and codes are available on Github (https://github.com/Shijia818/Tree-of-life) and from the Dryad Digital Repository: https://doi.org/10.5061/dryad.pzgmsbcnn [61]. The data are provided in electronic supplementary material [62].
